# Effects of Pr_6_O_11_ Addition on the Acid Resistance of Ceramic Proppant

**DOI:** 10.3390/ma10040427

**Published:** 2017-04-19

**Authors:** Guodong Xiong, Bolin Wu, Tingting Wu

**Affiliations:** College of Material Science and Engineering, Guilin University of Technology, Guilin 541004, Guangxi, China; xgd903040137@163.com (G.X.); 15207177716@163.com (T.W.)

**Keywords:** proppant, acid resistance, rare earth, solid solution, inorganic synthesis

## Abstract

This paper investigated the effect of Pr_6_O_11_ addition on the acid resistance of ceramic proppant. Acid resistance of proppants can be improved by introducing Pr_6_O_11_ into the Al_2_O_3_-CaO-MgO-SiO_2_ (ACMS) system. To illustrate and explain the mechanism of acid resistance, the samples were characterized by different techniques, using X-ray powder diffraction (XRD) and scanning electron microscopy (SEM). The phase structure of the specimens was characterized by XRD and SEM-detected microstructures of the specimens. It was observed that with the increase of rare-earth oxide content, the acid solubility of the specimens decreased, and then increased when it reached the minimum value 0.45 wt %. The results of the research show that the improvement of acid resistance with rare-earth oxides was achieved by refining the grain size, strengthening the grain boundary, and turning Ca_2_Al_2_SiO_7_, in which acid resistance is poor, into CaAl_12_O_19_, which possesses better acid resistance, and then enhance the acid resistance of the proppants. Furthermore, Pr_6_O_11_ can form a solid solution with Ca_2_Al_2_SiO_7_ and CaAl_12_O_19_. The acid resistance of CaAl_12_O_19_ improves with the increase of solid solubility. In contrast, the acid resistance of Ca_2_Al_2_SiO_7_ will decrease after Ca_2_Al_2_SiO_7_ forms a solid solution with Pr_6_O_11_.

## 1. Introduction

Facing the global energy crisis, many countries have to look for alternative energy sources. As one of the most important sources, shale gas, which is an unconventional gas contained in rock that must be broken open before the gas can flow easily to production wells, has already been well-developed during the past decade. The “shale” revolution started in the USA about 10 years ago, and the shale gas and oil revolution has unexpectedly and forcefully begun to change the landscape in the USA. The revolution is projected to extend around the world, leading to substantial economic and geopolitical impacts for energy-producing and -consuming nations [1]. Shale gas has become an increasingly important source of natural gas in the United State since the start of this century [2]. Recently, shale gas resources have been discovered in many countries [1,2,3]. With the rapid development of the gas industry in North American, the traditional pattern was broken, and a new market structure has been established. Hydraulic fracturing is known as the main and effective method for increasing oil and gas recovery and has made significant contributions to the petroleum industry [4]. It tends to increase the productivity of petroleum and gas wells by creating extensive artificial fractures around well bores so that the hydrocarbon production from the well can be significantly enhanced [4].

Fracturing proppants [5] are solid particles used in combination with fracturing fluid to keep the created fracture open during hydraulic fracturing [6]. Since the first fracturing operation was done with silica sand proppant in 1947, many materials have been used as proppants, including walnut hulls, natural sand, resin-coated sand, sintered bauxite, and fused zirconia. The proppant market report categorizes the global market by three types: sand, ceramic, and resin-coated proppant. Even now, sand and ceramic proppant are the two most common proppants in fracturing processes. In hydraulic fracturing operations they are transported with fracturing fluid at high pressure to the fractures underground over a distance of several kilometers. When the hydraulic pressure is removed, the proppants remain in the fractures and hold them open, improving the oil and gas fracture conductivity. Then gas and oil can flow to the wells fluently.

It is well known that investigations on the proppants started in 1947 and a series of works have been conducted by many scholars. Previous research works have focused on reducing the crushing rate and density, or improving strength [7,8,9]. However, there are few reports about studies on the acid resistance [10,11,12,13,14]. Since proppants must work underground over thousands of meters they have to endure a series of hash environments, such as high temperature, high pressure, and corrosion from various corrosive media in the crust or fracturing fluid. To maintain conductivity of channels under high pressure for a long time, structural failure must be avoided. If the proppant has poor resistance (chemical stability), it is easily crushed under high pressure [5,15,16]. After proppants are crushed, they produce fines that can plug the proppant pack and the porosity of the proppant is reduced, which subsequently reduces the permeability of the proppant pack. Permeability reduction in the proppant pack will, therefore, reduce the hydraulic fracture conductivity [17]. Obviously, to maintain their structural integrity in an acidic environment, acid resistance plays a critically important role in the fracturing process. 

Additionally, proppants with good acid resistance are necessary to maintain high fracture conductivity for a long time with the increasing drill hole depth. CARBO, as one of the largest manufacturers of ceramic proppants in the world, produces products that measure up in terms of world standard, and the acid solubility of their products is between 2.5 wt % and 4.5 wt %. Compared with CARBO, the best result is 0.45 wt % in our study by doping a trace of Pr_6_O_11_.

In our laboratory, many works about how to improve the acid resistance of ceramic proppant in the AMCS system by doping rare earths have been conducted. The purpose of this paper is to reveal the effects of doping with Pr_6_O_11_ on the acid resistance of ceramic proppant. This paper is focused on: (1) the effects on acid resistance by doping with Pr_6_O_11_; (2) the influences of Pr_6_O_11_ on grain growth and microstructure; and (3) revealing mechanisms about the improvement of acid resistance.

## 2. Experimental Process

Samples were prepared using commercial α-Al_2_O_3_ powder (99.8%, Quanzhou Huaming Trading Co. Ltd., Quanzhou, China) and CaO (99.0%, Xilong Chemical Industry Co. Ltd., Shantou, China) MgO (98.0%, Xilong Chemistry Factory Co. Ltd., Shantou, China), SiO_2_ (99.8%, Xilong Chemical Industry Co. Ltd., Shantou, China), and the rare earth oxide Pr_6_O_11_ was introduced as a sintering aid. Then the raw materials were weighed and mixed with each other. Subsequently, the mixture was homogenized with water by ball-milling for 36 h. After drying, the powder was pressed into pellets and sintered at a heating rate of 3 °C/min, for 1 h.

Ca_2_Al_2_SiO_7_ and CaAl_12_O_19_ were synthesized by the conventional solid-state reaction technique. Firstly, the raw materials were weighted according to Table 1 and mixed with each other. Then the mixture was homogenized with alcohol media in a mortar. After drying, it was milled into powder. After pelleting and drying, the samples were heated to 1375 °C and 1750 °C at the rate of 3 °C/min for 2 h, respectively. The tests of apparent density and acid solubility were evaluated by the Chinese Petroleum and Gas Industrial Standard (SY/T5108-2006). The Archimedes method was used to measure the density of the sintered sample, and calculated according to the following formula: ρ = *M*/*V*, where *M* is the weight of dry sample (g) and *V* is the volume of sample (cm^3^). An acid test was performed in acid solution (12 wt % HCl + 3 wt % HF) at 65 °C for 30 min. The testing process of acid solubility is as follows for each sample: add 5 ± 0.1 g sample (*M*_1_) to 100 mL acid solution (12 wt % HCl + 3 wt % HF) at 65 °C for 30 min. Second, remove the sample from the acid solution and wash with deionized water until the flushing fluid become neutral. Then, dry the sample in an oven at 105 °C for 2 h. Finally, weigh the dry sample (*M*_2_) which has been corroded. Acid solubility was determined: *S* = (*M*_1_ − *M*_2_)/*M*_1_ × 100%, where *S* is acid solubility, *M*_1_ is the weight of the sample before acid treatment, and *M*_2_ is the weight of the sample after acid treatment.

The phase evolution was investigated by XRD (X’Pert PRO, PANalytical B.V., Almelo, The Netherlands) and the microstructure of the sintered samples was observed by SEM (S-4800, Hitachi High-Technologies Corporation, Tokyo, Japan).

## 3. Results and Discussion

### 3.1. Acid Resistance of Proppant

As can be seen in Table 2, the acid solubility of samples decreases first, and then begins to increase with the increase of Pr_6_O_11_. The acid solubility of sample P1 exhibits the best result at 0.45 wt %. The results indicate that the enhancing of acid resistance can be achieved by doping with a small amount of Pr_6_O_11_. It follows that acid resistance of proppants can be improved by a proper addition of Pr_6_O_11_ and it provides a direction to make proppants with good acid resistance by adding traces of Pr_6_O_11_.

### 3.2. Phase Analysis

**Figure 1:**
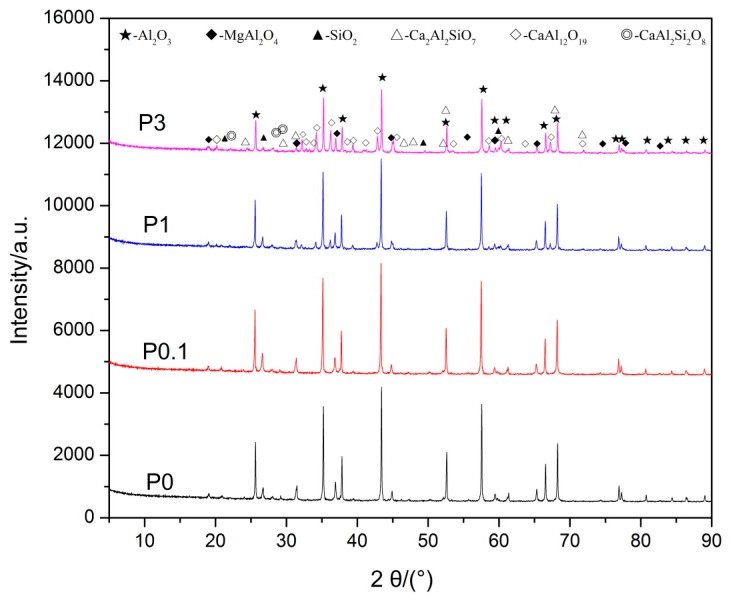
XRD patterns of proppant samples before acid treatment.

The results of XRD of samples P0, P0.1, and P1 are illustrated in Figure 1. The peaks of Al_2_O_3_, MgAl_2_O_4_, Ca_2_Al_2_SiO_7_, CaAl_2_Si_2_O_8_, and SiO_2_ (introduced when grinding in an agate mortar) were identified in Sample P0, whereas it is noted that there is a significant difference between sample P0 and P1, as observed in Figure 1. The Ca_2_Al_2_SiO_7_ phase disappeared and a new CaAl_12_O_19_ phase formed in sample P1. 

Table 2 shows that the apparent densities of all samples decrease gradually with an increase in the amount of Pr_6_O_11_. There is an obvious difference in the apparent density between sample P0 (3.67 g/cm^3^) and sample P1 (3.61 g/cm^3^). In general, the sample has less apparent density, leading to a poor acid resistance. However, the acid solubility of sample P1 did not increase; instead, it decreased obviously. Thus, it is considered that the results are caused by the generation of a new CaAl_12_O_19_ phase which comprises better acid resistance. Additionally, there is no phase which contains Pr ions that was detected by XRD, even when the content of Pr_6_O_11_ reached 3 wt %. It is possible that Pr_6_O_11_ formed a solid solution with grain boundary phases. 

In order to test the idea, Ca_2_Al_2_SiO_7_ and CaAl_12_O_19_ were synthesized and solid solution experiments of Pr^3+^ in Ca_2_Al_2_SiO_7_ and CaAl_12_O_19_ were conducted to simulate solid solution formation.

**Figure 2:**
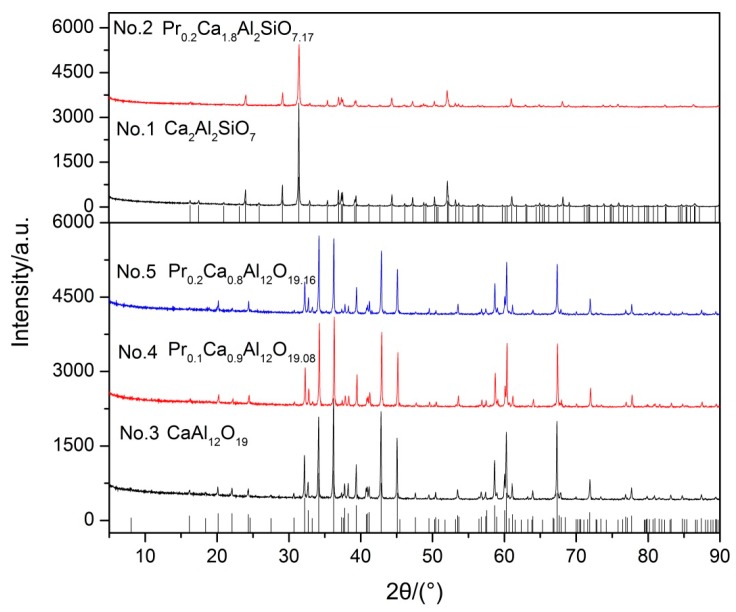
XRD patterns of synthetic samples.

In Figure 2, the XRD pattern shows that the diffraction peaks of all samples match well with their characteristic peaks, respectively. Furthermore, there are no impurities in all of the samples. The results indicate that Pr_6_O_11_ can form a solid solution with Ca_2_Al_2_SiO_7_ and CaAl_12_O_19_ in the sintering process. Subsequently, samples were pelleted, sintered, and then acid solubility tests were performed in acid solution (12 wt % HCl and 3 wt % HF) at 65 °C for 5 h. The results of acid solubility are shown in Table 1. Obviously, acid resistance of sample No. 1 (12.91 wt %) is poor compared with sample No. 3 (1.44 wt %) and CaAl_12_O_19_ comprises better acid resistance. In addition, we can observe that the acid solubility of CaAl_12_O_19_ decreases gradually with the increase of solid solubility according to Table 1. The results show that Pr_6_O_11_ not only turns Ca_2_Al_2_SiO_7_ into CaAl_12_O_19_, but also improves the acid resistance of CaAl_12_O_19_. In contrast, the acid resistance of Ca_2_Al_2_SiO_7_ is worsened after Ca_2_Al_2_SiO_7_ formed a solid solution with Pr_6_O_11_. Hence, on the basis of these experimental results we can demonstrate that acid resistance of different compounds decreases in the order of Pr_0.2_Ca_0.8_Al_12_O_19.16_ ˃ Pr_0.1_Ca_0.9_Al_12_O_19.08_ ˃ CaAl_12_O_19_ ˃ Ca_2_Al_2_SiO_7_ ˃ Pr_0.2_Ca_1.8_Al_2_SiO_7.17_. Our observations are consistent with Wu et al. [10], who reported that the improvement of acid resistance was caused by forming acid-resistance phases. In conclusion, Pr_6_O_11_ can prompt Ca_2_Al_2_SiO_7_ change into CaAl_12_O_19_, which possesses better acid resistance, and then enhance the acid resistance of proppants. Furthermore, acid resistance of CaAl_12_O_19_ can be enhanced by forming a solid solution with Pr_6_O_11_, but acid resistance of Ca_2_Al_2_SiO_7_ is worsened.

### 3.3. Microstructure Analysis

**Figure 3:**
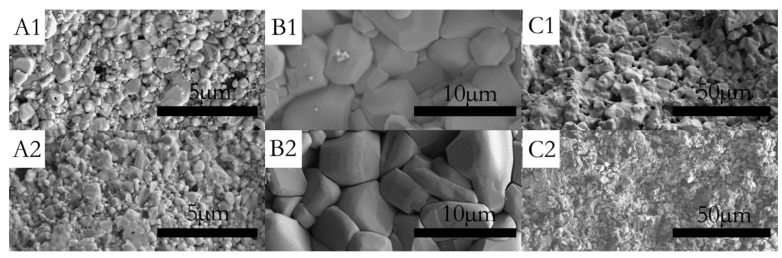
SEM micrograph of the surface (**A1**) sample P0 after acid treatment for 30 min; (**A2**) sample P0.1 after acid treatment for 30 min; (**B1**) sample No. 3 before acid treatment; (**B2**) sample No. 3 after acid treatment for 5 h; (**C1**) sample No. 1 before acid treatment; and (**C2**) sample No. 3 after acid treatment for 5 h.

**Figure 4:**
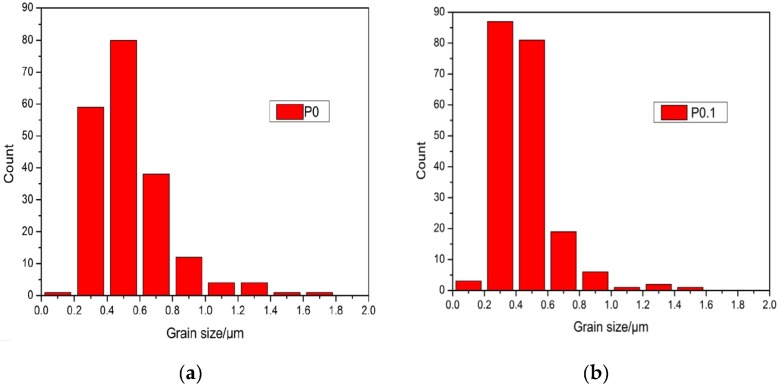
Grain size distribution of sample P0 and P0.1. (**a**) Grain size distribution of sample P0; (**b**) Grain size distribution of sample P0.1.

Figure 3A1,A2 show the surfaces of sample P0 and P0.1, which have been corroded for 30 min, respectively. Figure 3A2 shows that the grains of sample P0.1 are obviously refined and the diameter distribution became narrow by doping with Pr_6_O_11_. As can be seen in Figure 4, the grain size distribution of samples P0 and P0.1 are within sub-micron magnitudes, but the average size of sample P0.1 is smaller. The reasons for the increase of acid resistance by adding Pr_6_O_11_ are: at the upper temperature, rare earth ions mainly segregate to the grain boundaries in alumina, which block the diffusion of Al ions along grain boundaries. Rare earth ions play an important role in inhibiting grain growth by virtue of their influence on the grain boundaries’ migration during the sintering process, which can lead to the grains being obviously refined obviously and growing uniformly. Hence, sample P0.1, with a more compact structure, can prevent the acid solution from seeping. Finally, all of these factors resulted in the improvement of acid resistance simultaneously [18].

Figure 3B1,B2,C1,C2 show the surfaces of samples No. 1 and No. 3, which have been corroded for 5 h in acid solution. It is clear that sample No. 1 has been corroded seriously. The grain size of sample No. 1 decreases evidently after acid treatment and integrated structures of the grains have been destroyed by the acid solution. A loose structure can lead to high acid solubility. In contrast, the internal structure of CaAl_12_O_19_ was dense and without any corrosion signs.

## 4. Conclusions

In this paper, the effects of doping with Pr_6_O_11_ on the acid resistance of ceramic proppant were investigated. With the increase of Pr_6_O_11_, the acid resistance of samples increases gradually. The proppants in the ACMS system has good acid resistance, and the acid solubility is 0.45 wt % by doping a small amount of Pr_6_O_11_. As a result, the following conclusions can be offered:

1. The introduction of Pr_6_O_11_ in the ACMS system will restrict grain size development and form a fine-grained structure with low porosity so that the dense microstructure can protect proppants from being corroded badly by acid solution.
2. Adding Pr_6_O_11_ can change the type of phase, which turns Ca_2_Al_2_SiO_7_, in which acid resistance is poor, into CaAl_12_O_19_, which possesses better acid resistance, and enhances the acid resistance of the proppants.
3. Pr_6_O_11_ can form a solid solution with Ca_2_Al_2_SiO_7_ and CaAl_12_O_19_. The acid resistance of CaAl_12_O_19_ improves with the increase of solid solubility. In contrast, the acid resistance of Ca_2_Al_2_SiO_7_ will decrease after Ca_2_Al_2_SiO_7_ forms a solid solution with Pr_6_O_11_.

## Figures and Tables

**Table 1:** Chemical compositions and acid solubility of synthetic samples.

Samples No.	Stoichiometry	Chemical Composition (wt %)				Acid Solubility wt %
		Al_2_O_3_	CaO	SiO_2_	Pr_6_O_11_	
1	Ca_2_Al_2_SiO_7_	37.23	40.88	21.89	0	12.91
2	Pr_0.2_Ca_1.8_Al_2_SiO_7.17_	34.36	33.95	20.21	11.48	42.47
3	CaAl_12_O_19_	91.62	8.38	0	0	1.44
4	Pr_0.1_Ca_0.9_Al_12_O_19.08_	90.08	7.41	0	2.51	0.82
5	Pr_0.2_Ca_0.8_Al_12_O_19.16_	88.59	4.92	0	6.49	0.33

**Table 2:** Chemical compositions and some properties of proppant samples.

Sample Number	Chemical Composition (wt %)					Apparent Density g/cm^3^	Acid Solubility wt %	Sintering Temperature °C
	Al_2_O_3_	MgO	CaO	SiO_2_	Pr_6_O_11_			
P0	90	3.33	3.33	3.34	0	3.67	0.51	1375
P0.1	89.9	3.33	3.33	3.34	0.1	3.64	0.46	1350
P1	89	3.33	3.33	3.34	1	3.61	0.45	1375
P3	86	3.33	3.33	3.34	3	3.44	0.67	1450

## Supplementary Materials

The following are available online at www.mdpi.com/1996-1944/10/4/427/s1. Figure S1: SEM micrograph of the sample (A1) sample P0 after acid treatment for 30 min (A2) the internal structure of sample P0 (B1) sample P0.1 after acid treatment for 30 min (B2) the internal structure of sample P0.1.
