# Supplementary material for: Effects of Pr6O11 Addition on the Acid Resistance of Ceramic Proppant
Source: Materials (Basel). 2017 Apr 19;10(4):427. doi: 10.3390/ma10040427 (PMC5506917; doi:10.3390/ma10040427)
Supplement: Supplementary file 1 [file materials-10-00427-s001.pdf]

Supplementary Materials: Effects of Pr_6O_{11} Addition on the Acid Resistance of Ceramic Proppant

Guodong Xiong, Bolin Wu* and Tingting Wu

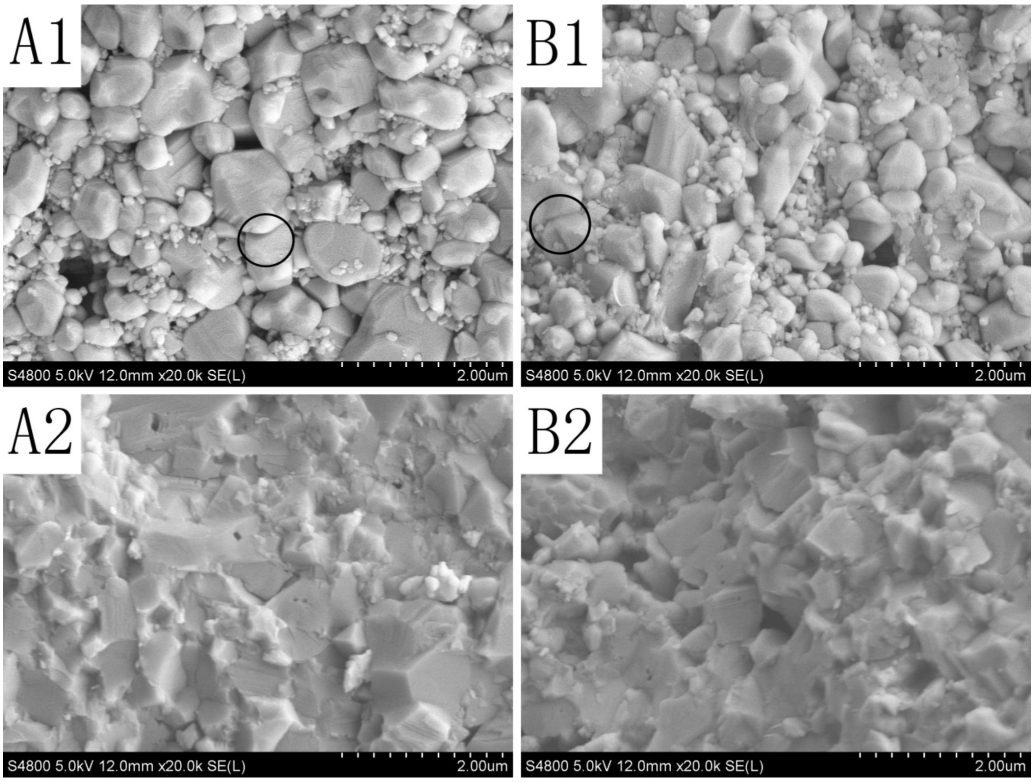

Figure S1. SEM micrograph of the sample (**A1**) sample P0 after acid treatment for 30 min (**A2**) the internal structure of sample P0 (**B1**) sample P0.1 after acid treatment for 30 min (**B2**) the internal structure of sample P0.1

As can be seen in Figure S1, the grain boundary cohesion of sample P0.1 is better compared with sample P0. And the fracture mode of alumina ceramics was changed by doping rare-earth oxides. A transgranular mode of fracture in alumina ceramics consumes more energy and generally leads to a higher K_{IC}(where K_{IC} is the fracture toughness) as compared with an intergranular fracture [1].

Reference:

1. Yao, Y.; Li, C.; Wang, L.; Jiang, X.; Qiu, T. Mechanical behaviors of alumina ceramics doped with rare-earth oxides. *Rare Metals* **2010**, *29*, 456–459.
